# Supplementary material for: Assessing the Occurrence of Host-Specific Faecal Indicator Markers in Water Systems as a Function of Water, Sanitation and Hygiene Practices: A Case Study in Rural Communities of Vhembe District Municipality, South Africa
Source: Pathogens. 2023 Dec 23;13(1):16. doi: 10.3390/pathogens13010016 (PMC10819538; doi:10.3390/pathogens13010016)
Supplement: Supplementary file 1 [file pathogens-13-00016-s001.zip › pathogens-2702104-supplementary.pdf]

Supplementary S1: Survey questionnaire

Date: ____________________

Name of Interviewer: ____________________

Name of Municipality and Village: ____________________

Household ID: ____________________

Language used for interview: ____________________

---

1. Socio-demography

1.1 Age			1.2 Gender		
1.3 Number of family members					
1.4 Adult female(s)		Adult male(s)		Children 17 years and below	
1.5 Age of members above	a.________ b.________ c.________				
1.6 How many family members are employed?					
1.7 How many family members have passed matric?					
1.8 How many family members have tertiary eductaion?					
1.9 What is the monthly household income?					
R5000 and less		Range between R5000-R10 000		R10 000 and more	
1.10 How much do you spend on household cleaning agents monthly?					

---

2. Water sources

2.1 What is the water source in your community?

Ponds		River/Stream		Dam		Other	
2.2 Is the water source protected from any trespassing animals?							
Yes		No					
2.3 If no, specify which animals?							
2.4 What is the source of potable water for your household?							
Tap in the yard		Municipal communal tap		Other			

2.5 Who fetches water for everyday use?							
Adult male		Adult female		Young male		Young female	

2.6 Do you engage in leisure activities at the river/dam?

Fishing		Swimming		Boat rides		No	

3. Storage of water

3.1 Distance of water source from household

Less than 10m		10-500m		500-1000m		1000m +	

.

3.2 Do you store the water?

Yes		No	

3.3 If yes, which containers do you use for water storage?

………..

3.4 How long is the water stored?

Less than a day		A few days		A week		Other	

3.5 Where are the water storage containers kept?

Inside the house		Outside the house	

3.6 How do you keep the containers?

Always open		Always closed	

3.7 How often do you wash the containers?

Never		Once a month		Before each collection/storage	

3.8 How do you withdraw water from the storage container?

By immersing a household utensil		Use storage container with tap	

Other: ……………………………………………………………………………………………………

3.9 Does your household get enough water for everyday use?

Yes		No	

3.10 If No, what is the reason for that?

………

4. Water treatment

4.1. Do you treat the water before usage?

Yes		No	

4.2 If Yes, how is the water treated?

Boiling		Bleaching		Alum stone		Other	

4.3 If No why?

……..……..

---

5. Source of faecal pollution

5.1 Do you have a toilet in the yard?

Yes		No	

5.2 If Yes, what type of sanitation facility is it?

Flushing		Pit latrine		VIP toilet		Bucket system	

Other: ………

5.3 If No, where do you relieve yourself?

Open area		In streams		Neighbour toilet		Communal toilet	

5.4 Does the sanitation facility have a handwash basin?

Yes		No	

5.5 Do you have animals around your water sources?

Yes		No	

5.6 If yes, which type of animals?

………

5.7 Do you have crops around your water sources?

Yes		No	

5.8 If yes, what type of crops?

………

5.9 Do children under the age of 5 use this toilet?

Yes		No	

5.10 If No; why not?

It’s not safe		It’s too far		It’s too dirty	

Other: ………………………………………………………………………………………………………

6. Health

6.1 When do you wash your hands?

After toilet use		After nappy change		After animal contact		Before food preparations		Before meals		After house chores and rubbish disposal	

Other……………………………………………………………………………………………………….

6.2 Do you use soap to wash hands?

Yes		No	

6.3 If No, why not?

Can’t afford it		Don’t see the point	

6.4 What are the frequently occurring health issues in the household?

Bilharzia (bloody urine)		Diarrhoea (loose stools)		Impetigo (Infectious skin rashes)		Worms in faeces	

6.5 If diarrhoea; does it happen to children under age 5?

Yes		No	
If Yes; how often?			

6.6 Does it occur in persons over 65?

Yes		No	
If Yes; how often?			

6.7 What is the nature of the stool of the infected person?

Watery		Mucopurulent		Bloody	

6.8 What is done with the sick person?

Nothing		Self-medicate		Traditional healer		Clinic/health centre	

6.9 Are there any quarantine measures for the convalescent individual?

Yes		No	

If Yes; explain briefly

……

Thank you!
